# First Aid and Pre-Hospital Management of Venomous Snakebites

**DOI:** 10.3390/tropicalmed3020045

**Published:** 2018-04-24

**Authors:** Jennifer Parker-Cote, William J. Meggs

**Affiliations:** Division of Toxicology, Department of Emergency Medicine, Brody School of Medicine at East Carolina University, Greenville, NC 27858, USA; parkercotej16@ecu.edu

**Keywords:** snakebites, first aid, emergency medicine services, pressure immobilization bandages

## Abstract

Background: Antivenom is the definitive treatment for venomous snakebites, but is expensive and not available in many rural and poorly developed regions. Timely transportation to facilities that stock and administer antivenom may not be available in rural areas with poorly developed emergency medical services. These factors have led to consideration of measures to delay onset of toxicity or alternatives to antivenom therapy. Methods: PubMed searches were conducted for articles on snakebite treatment, or that contained first aid, emergency medical services, tourniquets, pressure immobilization bandages, suction devices, and lymphatic flow inhibitors. Results: The reviewed articles describe how venoms spread after a venomous snakebite on an extremity, list the proposed first aid measures for delaying the spread of venoms, and evaluate the scientific studies that support or refute methods of snakebite first aid. The recommendations for field treatment of venomous snakebites will be discussed. Conclusions: The evidence suggests that pressure immobilization bandages and related strategies are the best interventions to delay onset of systemic toxicity from venomous snakebites but may increase local toxicity for venoms that destroy tissue at the site of the bite, so their use should be individualized to the circumstances and nature of the venom.

## 1. Introduction

Intravenous antivenom is the definitive treatment for venomous snakebites but is expensive, often in short supply, and not available in many rural and poorly developed regions [1,2]. Timely transportation to facilities that stock and administer antivenom may not be available in areas with poorly developed emergency medical services. These factors have led to consideration of measures to delay onset of toxicity or alternatives to antivenom therapy.

This review will describe how venoms spread after a venomous snakebite on an extremity, list the proposed first aid measures for delaying spread of venoms, and evaluate the scientific studies that support or refute methods of snakebite first aid. The recommendations for field treatment of poisonous snakebites will be discussed.

## 2. Methods

PubMed searches were conducted for articles on snakebite treatment, or that contained first aid, emergency medical services, tourniquets, pressure immobilization bandages, suction devices, and lymphatic flow inhibitors. The search encompassed articles from 1945 to 2017. This review emphasizes articles that initially proposed a technique and recent experimental studies that support or refute the technique.

### 2.1. Systemic Spread of Venom

A venomous snake can inject venom into subcutaneous tissues, muscle, a vein, or an artery. Intravascular injections result in rapid onset of systemic toxicity [3,4] and can be difficult to treat, even when antivenom is readily available. No first aid measure, other than supportive care and advanced life support, will be of benefit in intravascular venom injections.

In a classic study published in 1941, Barnes and Trueta [5] proved that lymphatic flow from an extremity is the main route via which venoms spread from a subcutaneous extremity injection, but demonstrated that systemic arrival of venom is delayed by lymphatic ligation. Rabbits were injected in a hind leg with a fatal dose of tiger snake (*Notechis scutatus*) venom—a potent neurotoxin. The treatment group had the groin lymphatic channels ligated. The endpoint was time to death from respiratory paralysis. Rabbits in the control group expired after an average time of ten minutes, while those with lymphatic channel ligation survived an hour. This historic study established that flow through the lymphatic channels was the major route for venoms to reach the systemic circulation. Further, the study suggested that methods to block lymphatic flow can be effective for delaying onset of toxicity.

There are four known mechanisms to propel lymph from an extremity to the systemic circulation. (a) Large lymphatic vessels have smooth muscles in their walls that contract, which is the primary mode of lymph propulsion in an extremity at rest, suggesting pharmacologic blockade of the intrinsic lymph pump can retard venom transport. (b) Contraction of skeletal muscles can also propel lymph to the central circulation, which suggests that immobilization of an extremity can retard lymph flow. (c) Lymph channels, like veins, have one-way valves that prevent backflow of lymph. (d) Another set of valves, the contractile valves of lymph endothelial cells, also prevent backward flow of lymph [6].

### 2.2. Tourniquets

Tourniquets are constricting bands that block arterial, venous, and lymphatic flow. Tourniquets are often used as first aid after snake envenomation, especially in rural areas where transport times to a healthcare facility are increased. There are significant complications from their use, which can result in worsening morbidity and mortality.

A prospective study in the Philippines of 36 hospitalized patients who developed neurotoxic symptoms after bites from *Naja naja philippinensis* reported 4 patients who developed complete respiratory paralysis requiring mechanical ventilation after removal of a tourniquet [7]. From these findings, the authors recommended slowly releasing already-applied tourniquets. Amaral et al., found no difference in plasma creatine kinase enzyme activity, partial thromboplastic time, plasma whole venom, crotoxin concentrations, and frequency of acute renal and respiratory failure, or deaths between those who applied a tourniquet and those who did not, after *Crotalus durissus* envenomation [8].

A prospective study in Nigeria revealed that patients who received any form of first aid had a longer length of stay compared to those who received no form of first aid, and that those who had used a tourniquet had a larger antivenom requirement [9]. One case report of death after the application of a tourniquet for 48 h occurred; the patient suffered from thrombophlebitis, local necrosis, and gas gangrene, followed by pulmonary thromboembolism [10].

The lymphatic system transports venom from the envenomation site to systemic circulation. Application of a tourniquet will sequester venom locally, potentially leading to increased local tissue destruction when the venom is from those snakes that cause local tissue damage. Additionally, tourniquets can impede venous blood flow and arterial blood flow, leading to limb ischemia, gangrene, and potentially amputation.

Guidelines do not support the use of tourniquets. According to World Health Organization recommendations, arterial tourniquets are contraindicated; their effectiveness relies on occlusion of peripheral pulses causing pain, ischemia, nerve injury, and gangrenous limbs (level of evidence: expert opinion/consensus) [1,2]. Deleterious effects from tourniquets are seen within 20 min to 2 h of application. The Wilderness Medical Society does not recommend tourniquets for envenomations by pit vipers in North America [11].

### 2.3. Venom Extractors

Venom extractors are suction devices that are proposed to work by applying suction to the site of a snakebite. These devices are marketed by companies that target outdoorsmen. The purported design is supposed to apply negative pressure over fang marks to induce venom extraction. Venom extractors can still be purchased through major retailors online and instores, despite several studies proving their ineffectiveness [12,13]. A human study by Alberts et al., modeling western diamondback envenomation, demonstrated only a mean 2.0% decrease in total body venom load and an average 0.04% decrease of mock venom at the site of envenomation after 15 min of extraction. A porcine model with *Crotalus atrox* venom, by Bush et al., demonstrated concerns for injury by the extraction device and no benefit in decreasing local tissue injury after 30 min of extraction [13]. Alberts et al. suggested that the extractors could have collapsed the subcutaneous tissue, and pulled fluid from superficial capillaries that contain minimal venom load [12]. Our opinion is that any significant change in clinical outcomes is doubtful with such minute changes in venom load.

Proponents of extractors may counter with the difficulty in replicating a true envenomation with simulated venom or in an animal model. Single puncture sites instead of two puncture sites were used in the human study to decrease harm to patients, which is a less common presentation of snake envenomation [12]. Other variables that are difficult to replicate in human studies are the specific venom components, and the mechanism of action varies among species of snakes. Also, the volume of venom typically increases with the size of the snake.

At the time of this review, there are two abstracts that support the suction device, but neither has been peer reviewed and they include only limited descriptions of methodology. A rabbit study using the Sawyer Extractor^®^ to retrieve radiolabeled iodine-125 southern Pacific rattlesnake (*Crotalus viridis helleri*) venom reportedly removed 23% of the venom within the first 3 min, and 34% after 30 min of extraction [14]. The second abstract assessed the use of the Sawyer Extractor^®^ pump in 3 individuals envenomated by western diamondback rattlesnakes (*Crotalus atrox*) [15]. The volume of fluid extracted from the victims’ wounds after the initial application of the extractor did not yield a significant concentration to reduce toxicity. This small amount extracted is unlikely to be of clinical significance.

There are risks with use of these devices: local tissue destruction and a false sense of security. Bush et al. noted necrosis of tissue with resultant tissue loss in two animals treated with extraction, which was not seen in untreated animals [13]. Some have alleged that manufacturer claims provide a ‘false sense of security,’ and may delay a patient from seeking definitive and effective care [16]. Instructional inserts on the Sawyer Products B4 Extractor Pump Kit^®^ (Sawyer Products, Safety Harbor, FL, USA) do provide a warning that ‘The pump is not a replacement for quickly seeking professional medical treatment.’

Current guidelines from the Wilderness Medical Society and American Heart Association do not support the use of mechanical suction for pit viper envenomations [7,17]. An initial search of the medical literature did not yield results addressing the use of suction for other types of snake envenomations, such as elapids. Despite the evidence against use of mechanical suction, there are many unreliable sources available to the public. One study found that of 48 websites reviewed for accuracy of information regarding pre-hospital care of snakebites, 26 (54.1%) contained inappropriate recommendations [18]. Education for clinicians and the public regarding safe and effective pre-hospital care is imperative in preventing further harm to snakebite victims.

### 2.4. Electric Shock

Electric shock has been advocated as a treatment for venomous snakebites, though there is no scientific rationale for use of electric shock and no data to support its use, other than case reports. Its use was advocated in a letter to the editor of Lancet in 1986, in which it was claimed that the experience in the jungles of Ecuador of applying a high-voltage, low-amperage electric current to the grounded extremity prevented both local and systemic toxicity [19]. Electric shocks have been administered from outboard motors and stun guns. It was ineffective in a controlled study of mice injected with northern Pacific rattlesnake (*Crotalus virldis oreganus*) venom at varying doses [20]. The shocks neither reduced toxicity in rats injected with sublethal doses of fer-de-lance (*Bothrops atrox*) venom nor lethality in mice injected with lethal doses [21]. In a case report, administration of electric shock resulted in coma and incontinence [22]. The United States Food and Drug Administration has banned the use of these devices, which were previously sold to the public for the treatment of snakebites and spider bites (United States of America vs. CONO).

### 2.5. Pressure Immobilization Bandages

The pressure immobilization bandage consists of an ace wrap and splint and is known as the Commonwealth Serum Laboratories Method. A properly applied ace wrap is sufficient pressure to block lymphatic flow to an extremity while preserving arterial and venous flow. The splint prevents muscle contraction that squeezes lymph toward the central circulation. The result is to sequester venom in the extremity, which is reasonably expected to delay systemic toxicity. For venoms that cause local necrosis, local damage at the bite site could increase while necrosis could spread away from the bite site.

A study published in 1974 [23] injected monkeys with tiger snake (*Notechis scutatus*) venom and monitored plasma venom levels by radioimmunoassay. Treatment was immobilization of the extremity with a splint and the application of a firm crepe bandage to the length of the extremity. This intervention resulted in low levels of circulating venom relative to controls. Neither application of a splint alone nor of the pressure bandage alone delayed systemic detection of venom.

**Figure 1:**
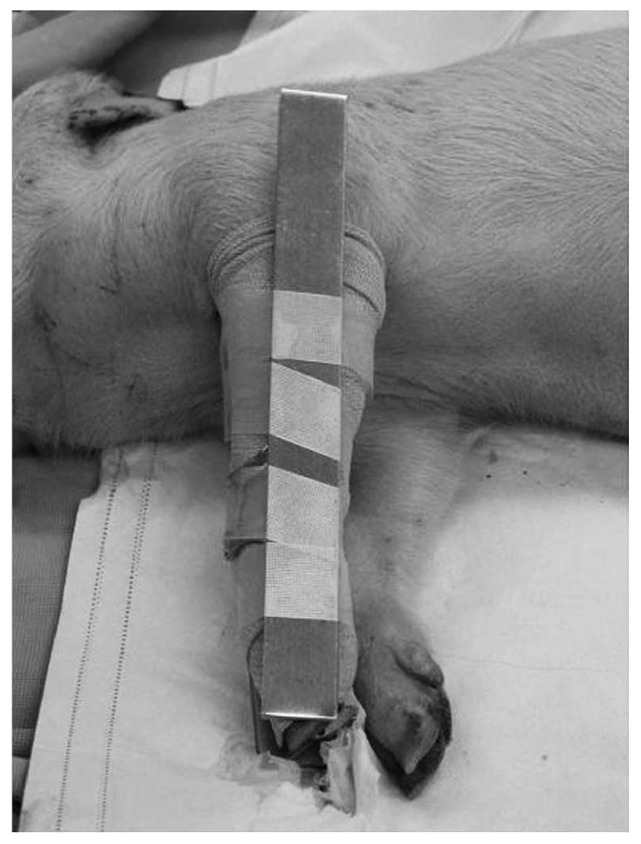
Pressure immobilization bandage consisting of an ace bandage wrapped beginning at the site of bite and wrapping the entire extremity, followed by a splint, in a porcine model of eastern coral snake envenomation. From [24], used with permission of the publisher.

**Figure 2:**
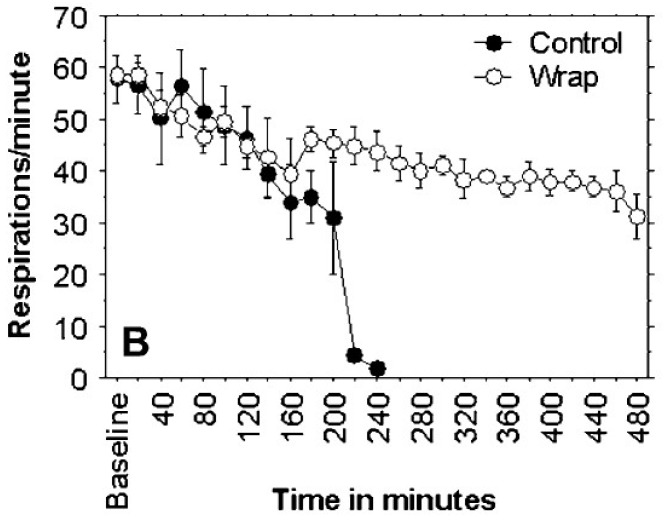
The pressure immobilization bandage in Figure 1 delayed onset of respiratory depression in treated pigs (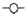) relative to controls (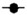) as seen in the plot of respiratory rate versus time. From [24], used with permission of the publisher.

A controlled study of pressure immobilization bandages (Figure 1) in anesthetized pigs injected with eastern coral snake (*Micrurus fulvius fulvius*) venom had an endpoint of survival to eight hours and used histological analysis of biopsies of the injection site [24]. Four of five subjects in the treatment group survived to 8 h, while no control group subjects survived. Mean time to onset of respiratory compromise was 170.4 ± 33.3 min in the control group (Figure 2). None of the pigs had histologic changes at the envenomation site consistent with ischemia or necrosis.

This result motivated a study of the long-term efficacy of a pressure immobilization bandage for coral snake (*Micrurus fulvius fulvius*) [25] in the porcine model. The hypothesis was that by sequestering the venom in the extremity with pressure immobilization for an extended period, the venom would be degraded in time by local processes such as hydroxylation. A randomized, controlled observational pilot study was conducted with ten pigs. Subjects were anesthetized, intubated, and injected subcutaneously with 10 mg of lyophilized coral snake (*Micrurus fulvius*) venom suspended in 1 mL of water. Treatment was a compression bandage and fiberglass cast applied 1 minute after envenomation. Pigs were monitored daily for 21 days for signs of respiratory depression, decreased oxygen saturations, and paralysis. In case of respiratory depression, pigs were humanely euthanized and time to death recorded. Median survival time of control animals was 307 min compared with 1172 min in treated animals (*p* = 0.10). Sixty percent of pigs in the treatment group survived to 24 h versus 0% of control pigs (*p* = 0.08). Two of the treatment pigs survived to the end point of 21 days but had necrotic lesions of the distal extremity, which could have been from mechanical trauma from the splint rather than necrotic effects of the venom.

Pressure immobilization bandages for snakebites with local necrosis are suspected to increase local damage and are generally not recommended. Despite this admonition, experimental studies have been performed. A study was performed of eastern diamondback (*Crotalus adamanteus*) toxicity in monkeys injected subcutaneously with 6 mg of venom [26]. Venom levels in urine and plasma were measured by solid-phase radioimmunoassay. Plasma levels as high as 1300 ng/mL occurred within 15 min of injection in controls, with progressive swelling in the injected limb. Firm pressure to the injection site and immobilization of limb with a splint were effective in keeping venom levels low until first aid was discontinued. The first aid measures prevented swelling. Animals treated with the first aid measures plus antivenom had less toxicity relative to those treated with antivenom alone, particularly when first aid was given prior to removal of the pressure immobilization bandage and splint. The authors recommended based on their study that the use of pressure immobilization bandages be considered for humans bitten by *C. adamanteus*.

A pilot study of pressure immobilization bandages for rattlesnake bites was also performed in the porcine model using a large dose of western diamondback rattlesnake (*Crotalus atrox*) venom [27]. A dose of 200 mg of lyophilized venom was chosen to test the concept, because this is the maximum amount that is obtained from a single milking of this species. A randomized controlled study was conducted with six pigs. The ace bandage and splint were applied immediately after subcutaneous administration of venom in a distal hind paw. Endpoint was survival to 24 h. Three subjects in the control group expired between 191 and 305 min. All subjects in the treatment group survived the 24 h period. Antivenom was administered to the subjects in the treatment group prior to removal of the bandage; however, one subject expired after the bandage was removed with hyperkalemia, most likely from cell necrosis from the venom. While local necrosis did occur and required treatment with analgesia and antibiotics, subjects who survived were walking on the extremity 7 days after envenomation.

### 2.6. Compression Pad and Ring

A compression pad, also known as the Monash Method, consists of a pad placed over the site of envenomation and held in place with a nonelastic band with a pressure of at least 70 mm Hg, combined with a splint to immobilize the extremity. This method was tested in humans with a radiolabeled mock venom with comparisons to an air splint and a pressure immobilization bandage [28]. The air splint and pressure immobilization bandage were used with pressure adjusted to 55 mm Hg. Mock venom was measured in the serum. Venom in the serum was only retarded relative to controls by the pressure pad at 70 mm Hg. Whether or not this difference was due to the difference in devices or the increased pressure used for the compression pad is not known.

A prospective study using the compression pad was conducted in 15 subjects bitten by the Russell’s viper (*Daboia russelii siamensis*) in Myanmar. Serum venom levels were monitored after the application of a rubber pad placed over the bite site and attached with cotton bandages. The rubber pad measured 65 × 65 × 25 mm and the cotton bandage measured 65 mm × 1.6 m, except for bites over the fingers and toes, for which measurements were 55 × 30 × 25 mm and 55 × 250 mm for the pad and bandage, respectively. Proof that the pad retarded venom reaching the central circulation was demonstrated because venom levels remained constant in 15 of 23 subjects from the time the pad was placed until the pad was removed. After the pad was removed, venom levels increased by 10 to 40 ng/mL. In the remaining 7 subjects, serum venom levels became undetectable while undergoing the pad treatment [29].

**Figure 3:**
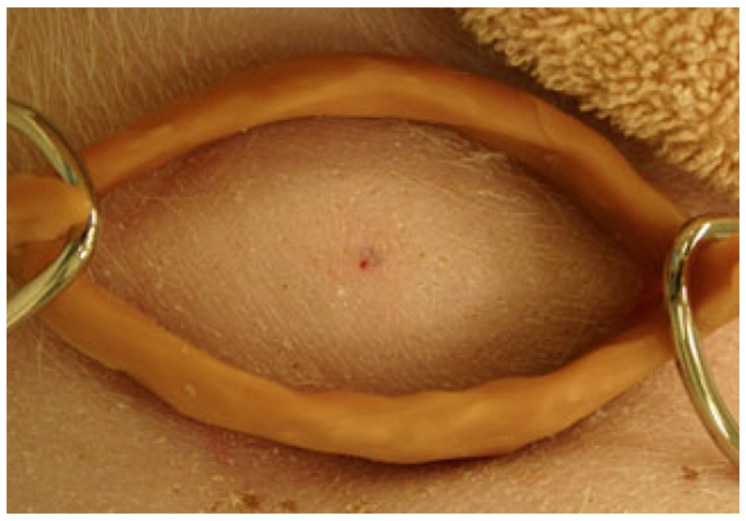
Circumferential device applied with bands to inhibit lymphatic flow from a torso envenomation. From [30], used with permission of the publisher.

Since pressure immobilization bandages could not be used for bites on the torso, consideration was given to a circumferential compression device consisting of a ring with two hooks for attaching a band to apply pressure (Figure 3). This device was tested in the porcine model for experimental torso envenomations for both eastern coral snake (*Micrurus fulvius fulvius*) [30] and eastern diamondback rattlesnake [31].

The device consists of a ring with two hooks to attach an elastic belt. The dimensions were 8 × 5 × 3 cm. It was secured with an elastic belt wrapped around the animal. For the coral snake study, a subcutaneous injection of a 10 mg lethal dose was injected in the torso. Endpoint was survival to 8 h. Five of the six pigs in the treatment group survived 8 h, with one pig expiring after 293 min. The three pigs in the control group developed respiratory failure at 322 min (range 272 to 382 min). The result was significant, with Fisher’s exact test value of 0.04.

In the test of the compression ring device for the eastern diamondback rattlesnake experimental envenomation to the torso in the porcine model, 50 mg of lyophilized venom reconstituted in sterile water was injected subcutaneously in sedated subjects allowed to breathe spontaneously. Subjects were monitored for cardiovascular collapse, fatal arrhythmia, loss of mean arterial pressure or pulse, or respiratory arrest. Subjects in the treatment group had mean a time to toxicity of 355 ± 65 min compared with a time of 32 ± 3.5 min in the control group, a result that was significant using a paired *t*-test (with *p* < 0.03).

A study of pressure immobilization for intramuscular injections in a porcine model of western diamondback rattlesnake (*Crotalus atrox*) envenomation found that pressure immobilization prolonged survival and produced less edema than that observed in control animals, but increased intracompartment pressures relative to in control animals without pressure immobilization [32].

### 2.7. Lymphatic Flow Inhibitors

Lymphatic flow inhibitors are pharmaceuticals that function by releasing nitric oxide, a compound that inhibits the intrinsic lymphatic pump. Hence, nitric oxide releasing agents have been considered as treatment to delay toxicity from venomous snakebites. Once such agent, glyceryl trinitrate ointment (GTNO), has been studied as a topical agent in both humans and rats injected with mock venom. GTNO (0.2% weight/weight) is commercially available (Rectogesic, Care Pharmaceuticals, Bondi Junction, Australia). Human volunteers (6 male, 9 females, age range 20 to 65 years) were injected in the feet with 50 microliters of sterile radiolabeled colloid. A crossover design was used, with each subject having venom injected with and without treatment. Foot to groin transit time of the mock venom time increased from 13 min (range 4 to 81 min) in the controls to 54 min (range 6.5 to 162 min) with treatment. The result was reported as highly significant with *p* < 0.0001 (statistical method not specified [33]). The treatment was also studied in anesthetized rats injected in the hind foot with eastern brown snake venom (*Pseudonaja textilits*). The experiment demonstrated that smearing the entire extremity with GTNO increased the time to respiratory arrest from 65 ± 4 min in control rats to 96 ± 6 min in GTNO-treated rats (*p* < 0.001; log-rank test) [33].

### 2.8. Trypsin Injection

Trypsin is a proteolytic enzyme that has the potential to degrade protein toxins. The proposed mechanism of action is to digest protein venoms. A randomized, blinded study in anesthetized pigs was conducted [34]. One minute after injection in a distal hind limb of 10 mg of eastern coral snake venom (*Micrurus fulvius fulvius*) dissolved in 1 mL of water, subjects were randomized to receive either 1 mL of saline or 1 mL of trypsin (100 mg/mL) at the envenomation site by a blinded investigator. Endpoint was survival to 3 days. Respiratory depression occurred more frequently in control subjects than in those that received trypsin (*p* = 0.009; Fisher’s exact test). Four of the six pigs that received trypsin survived to the end of the 3-day study while no control pigs survived. Trypsin is inexpensive and readily available and would most likely be used for highly potent protein venoms, but further study is needed before trypsin could be recommended for general use.

### 2.9. Herbal Medicines

Medicinal plants are used to treat snakebites. A large volume of literature exists on the topic that is too large to review in this article. A recent review focused on their use in Central America [35] and concluded that ‘available pharmacological data suggest different plant species may target different symptoms of snakebites, such as pain or anxiety, although more studies are needed to further evaluate the scientific basis for their use.’ A study in Brazil [36] found that extracts of the plant *Jatropha mollissima* (Euphorbiaceae), used in folk medicine, had efficacy in reducing local effects from snakebites by the *Bothrops* genus that cause the majority of snakebites in Brazil, and suggested that it be used as an adjuvant to antivenom, which the authors report is less effective in reducing local effects than systemic effects. A study in a mouse model of *Bothrops* envenomation found that the plant *Costus spicatus*, used in folk medicine as an anti-inflammatory medication, reduced pain and inflammation [37]. *Euphorbia hirta* was found to reduce lethality in a mouse model of Indian cobra (*Naja naja*) experimental snakebites, and the compound quercetin-3-O-alpha-rhamnoside extracted from the plant was found to have activity against the venom [38]. These and many other studies suggest that traditional medicinal plants reduce pain and suffering from snakebites and, in specific cases, may prevent lethality. Further study in this area could lead to specific pharmaceuticals targeted to species against which they have activity, though development in this area is expensive and unlikely to be widely funded.

### 2.10. Position Statements

According to the World Health Organization Guidelines for the Management of Snake-Bites [1],

Most of the familiar methods for first-aid treatment of snake-bite, both western and ‘traditional/herbal’, have been found to result in more harm (risk) than good (benefit). Their use should be discouraged and they should never be allowed to delay the movement of the patient to medical care at the hospital or dispensary. Recommended first-aid methods emphasize reassurance, immobilization of the whole patient and particularly the bitten limb and movement of the patient to a place where they can receive medical care as soon as possible.

A joint position statement by the American College of Medical Toxicology, American Academy of Clinical Toxicology, American Association of Poison Control Centers, European Association of Poison Centers and Clinical Toxicology, International Society of Toxonology, and the Asian Pacific Association of Medical Toxicology concluded [39],

Given that the primary toxic effect of envenomation is local tissue injury, mortality is not an ideal outcome measure to extrapolate to human crotaline envenomation. Available evidence fails to establish the efficacy of pressure immobilization in humans, but does indicate the possibility of serious adverse events arising from its use. The use of pressure immobilization for the pre-hospital treatment of North American Crotalinae envenomation is not recommended.

The American Heart Association first aid manual (combined 2010 and 2015 recommendations) states that the use of pressure immobilization bandages for venomous snakebites be considered, stating [17],

Applying a pressure immobilization bandage with a pressure between 40 and 70 mm Hg in the upper extremity and between 55 and 70 mm Hg in the lower extremity around the entire length of the bitten extremity is a reasonable way to slow the spread of venom by slowing lymph flow.

For practical purposes pressure is sufficient if the bandage is comfortably tight and snug but allows a finger to be slipped under it. Initially it was theorized that slowing lymphatic flow by external pressure would only benefit victims bitten by snakes producing neurotoxic venom, but the effectiveness of pressure immobilization has also been demonstrated for bites by non-neurotoxic American snakes in an animal model. The challenge is to find a way to teach the application of the correct snugness of the bandage because inadequate pressure is ineffective and too much pressure may cause local tissue damage. It has also been demonstrated that, once learned, retention of the skill of proper pressure and immobilization application is poor.

In Australia, where there are neurotoxic elapid snakes, the recommendation for snakebite first aid is pressure immobilization bandages [40]:

The first aid for a suspected or definite snake bite is a pressure bandage with immobilization (PBI). The pressure bandage should be a broad (15 cm) elastic bandage, rather than a crepe bandage. The bandage is applied over the bite site and then distally to proximally covering the whole limb. It should be applied about as tight as that used for a sprained ankle. The limb and whole patient should be immobilized for the first aid to be effective. The bandage and immobilization should remain until the patient has been transferred and assessed in hospital. The bandage should only be removed if antivenom is available and after there is no evidence of envenoming based on the admission laboratory tests and clinical examination. If the patient is envenomed the bandage can be removed after antivenom has been administered.

## 3. Conclusions

Antivenom is the definitive treatment for venomous snakebites and should be administered as soon as possible after a bite. First aid measures should be directed at reducing systemic toxicity by limiting lymphatic flow. Splints, rest, and avoidance of movement should reduce movement of the involved extremity. Positioning of the extremity below or at the level of the heart should be individualized—for snakebites with severe and potentially fatal systemic toxicity, systemic toxicity might be delayed by positioning the extremity below the heart, while for snakebites with severe local tissue damage and less systemic toxicity, positioning the extremity below the heart could increase local toxicity.

Pressure immobilization bandages, compression pads, and compression rings have been shown in experimental studies to delay systemic absorption of venom and to reduce mortality in some models. Limited human data supports their use in specific circumstances. They have not been as effective in field use relative to the laboratory settings because bandages have been applied too loosely, have not been applied to the entire extremity, and use of a splint has been inconsistent [41]. In a simulated setting, neither health professionals nor members of the general public performed well in applying pressure immobilization bandages [42]. The materials necessary for applying this and other techniques may not be readily available in many circumstances. Their use should be individualized to the circumstances of the bite, including region, species, timeliness of definitive treatment, and local guidelines. They are expected to be most helpful in snakebites with lethal systemic toxicity, limited local toxicity, and a long delay to definitive treatment with antivenom.

Pharmacological treatment with the topical nitric oxide inhibitor GTNO and injection of trypsin delayed toxicity in experimental studies but need further study. At this time, it is not possible to give a recommendation for the use of GTNO. Injection of trypsin at the site of the bite has likewise been efficacious in the treatment of eastern coral snake envenomations in an experimental study but is not sufficiently developed to recommend its use at this time. Plant extracts used in traditional medicine have been shown to help with pain and inflammation, and in certain cases to reduce lethality in experimental studies, and can be considered in rural areas in which folk remedies are available, but not antivenom.

Injection of trypsin at the site of envenomation had efficacy in one experimental study in a porcine model and could conceivably play a role in selected situations. However, this approach needs further investigation.

A number of treatments are generally not recommended. Tourniquets cause limb ischemia and amputations. Electric shock is dangerous in experimental studies and is not recommended. Suction devices have no efficacy in experimental studies and cannot be recommended.
